# Benzamidinium tetra­hydro­penta­borate sesquihydrate

**DOI:** 10.1107/S1600536808017522

**Published:** 2008-06-19

**Authors:** Gustavo Portalone

**Affiliations:** a Chemistry Department, University of Rome "La Sapienza", P.le A. Moro 5, I-00185 Rome, Italy

## Abstract

The asymmetric unit of the title compound [systematic name: benzamidinium 3,3′,5,5′-tetra­hydr­oxy-1,1′-spirobi[2,4,6-trioxa-1,3,5-triboracyclo­hexa­ne](1−) sesquihydrate], C_7_H_9_N_2_^+^·B_5_H_4_O_10_^−^·1.5H_2_O, is composed of two protonated benzamidinium cations, two tetra­hydro­penta­borate anions and three water mol­ecules of crystallization. The ions and water molecules are associated in the crystal structure by an extensive three-dimensional hydrogen-bonding network, which consists mainly of cation-to-anion N—H⋯O and anion-to-anion O—H⋯O hydrogen bonds.

## Related literature

For crystal structure determinations of the tetra­hydro­penta­borate anion, see: Loboda *et al.*, (1993 ▶, 1994 ▶); Wiebcke *et al.* (1993 ▶); Turdybekov *et al.* (1992 ▶); Freyhardt *et al.* (1994 ▶); Baber *et al.* (2004 ▶). For the computation of ring patterns formed by hydrogen bonds in crystal structures, see: Etter *et al.* (1990 ▶); Bernstein *et al.* (1995 ▶); Motherwell *et al.* (1999 ▶). For hydration in mol­ecular crystals, see: Gillon *et al.* (2003 ▶).
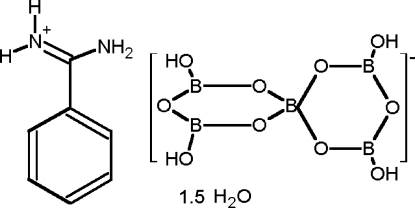


## Experimental

### 

#### Crystal data

C_7_H_9_N_2_^+^·B_5_H_4_O_10_^−^·1.5H_2_O
*M* *_r_* = 366.27
Triclinic, 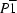

*a* = 8.22314 (19) Å
*b* = 10.7814 (2) Å
*c* = 19.1997 (3) Å
α = 75.9475 (11)°
β = 85.4458 (16)°
γ = 73.6979 (13)°
*V* = 1584.74 (5) Å^3^
*Z* = 4
Mo *K*α radiation
μ = 0.14 mm^−1^
*T* = 298 (2) K
0.15 × 0.12 × 0.10 mm

#### Data collection

Oxford Diffraction Xcalibur S CCD diffractometer
Absorption correction: multi-scan (*CrysAlis RED*; Oxford Diffraction, 2006 ▶) *T*_min_ = 0.975, *T*_max_ = 0.988
142668 measured reflections
9063 independent reflections
5688 reflections with *I* > 2σ(*I*)
*R*_int_ = 0.050

#### Refinement

*R*[*F* ^2^ > 2σ(*F* ^2^)] = 0.041
*wR*(*F* ^2^) = 0.098
*S* = 0.99
9063 reflections
542 parameters
1 restraint
H atoms treated by a mixture of independent and constrained refinement
Δρ_max_ = 0.23 e Å^−3^
Δρ_min_ = −0.22 e Å^−3^

### 

Data collection: *CrysAlis CCD* (Oxford Diffraction 2006 ▶); cell refinement: *CrysAlis RED*(Oxford Diffraction 2006 ▶); data reduction: *CrysAlis RED*; program(s) used to solve structure: *SIR97* (Altomare *et al.*, 1999 ▶); program(s) used to refine structure: *SHELXL97* (Sheldrick, 2008 ▶); molecular graphics: *ORTEP-3* (Farrugia, 1997 ▶); software used to prepare material for publication: *WinGX* (Farrugia, 1999 ▶).

## Supplementary Material

Crystal structure: contains datablocks global, I. DOI: 10.1107/S1600536808017522/rz2220sup1.cif

Structure factors: contains datablocks I. DOI: 10.1107/S1600536808017522/rz2220Isup2.hkl

Additional supplementary materials:  crystallographic information; 3D view; checkCIF report

## Figures and Tables

**Table 1:** Hydrogen-bond geometry (Å, °)

*D*—H⋯*A*	*D*—H	H⋯*A*	*D*⋯*A*	*D*—H⋯*A*
O2—H2⋯O3^i^	0.83 (2)	2.04 (2)	2.8562 (13)	167.4 (18)
O4—H4⋯O6*A*^ii^	0.87 (2)	2.00 (2)	2.8361 (13)	161.3 (18)
O7—H7⋯O5*A*^iii^	0.89 (2)	1.80 (2)	2.6877 (13)	177 (2)
O9—H9⋯O1*A*^iv^	0.87 (2)	1.91 (2)	2.7784 (14)	174.6 (19)
O2*A*—H2*A*⋯O10^iv^	0.87 (2)	1.84 (2)	2.7050 (14)	174.5 (17)
O4*A*—H4*A*⋯O6^iii^	0.89 (2)	1.79 (2)	2.6735 (13)	178 (2)
O7*A*—H7*A*⋯O5^ii^	0.92 (2)	1.93 (2)	2.8085 (15)	160 (2)
O9*A*—H9*A*⋯O2*W*	0.87 (2)	2.18 (2)	2.9474 (16)	147.4 (18)
N1—H11⋯O1*W*	0.870 (18)	2.059 (18)	2.8756 (18)	156.0 (16)
N1—H12⋯O2*A*^v^	0.872 (18)	1.996 (18)	2.8484 (15)	165.7 (15)
N2—H21⋯O1*W*	0.893 (17)	2.330 (17)	3.0892 (18)	142.8 (14)
N2—H22⋯O4*A*	0.858 (17)	2.025 (18)	2.8772 (16)	172.0 (15)
N1*A*—H11*A*⋯O10*A*	0.869 (19)	2.238 (19)	3.0084 (18)	147.7 (16)
N1*A*—H12*A*⋯O8	0.892 (19)	2.12 (2)	2.9646 (17)	157.4 (16)
N2*A*—H21*A*⋯O2*W*^iv^	0.86 (2)	2.01 (2)	2.8703 (18)	175.0 (18)
N2*A*—H22*A*⋯O9	0.891 (19)	2.181 (19)	3.0717 (16)	178.4 (16)
O1*W*—H11*W*⋯O1^vi^	0.82 (2)	2.10 (2)	2.9180 (15)	173 (2)
O1*W*—H12*W*⋯O4^vii^	0.85 (2)	2.22 (2)	3.0033 (18)	153 (2)
O2*W*—H21*W*⋯O3*W*	0.93 (2)	1.92 (2)	2.8199 (19)	163 (2)
O2*W*—H22*W*⋯O7^viii^	0.91 (2)	1.90 (2)	2.8016 (14)	169.3 (19)
O3*W*—H31*W*⋯O3*W*^ix^	0.72 (3)	2.488 (10)	3.003 (3)	130.5 (17)
O3*W*—H32*W*⋯O7*A*	0.98 (3)	2.05 (3)	2.983 (2)	159 (2)

Symmetry codes: (i) 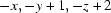; (ii) 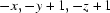; (iii) 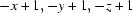; (iv) 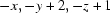; (v) 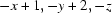; (vi) 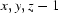; (vii) 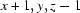; (viii) 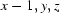; (ix) 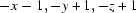.

**Fig. 1.**
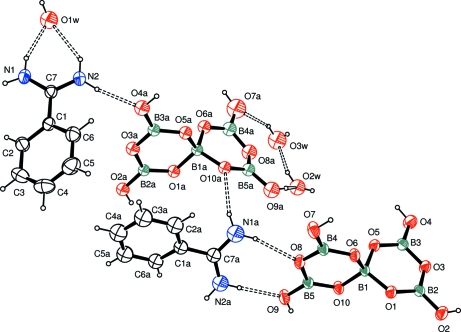
The molecular structure of the title compound, showing the atom-labelling scheme. Displacements ellipsoids are at the 50% probability level. Hydrogen bonding is indicated by dashed lines.

**Fig. 2.**
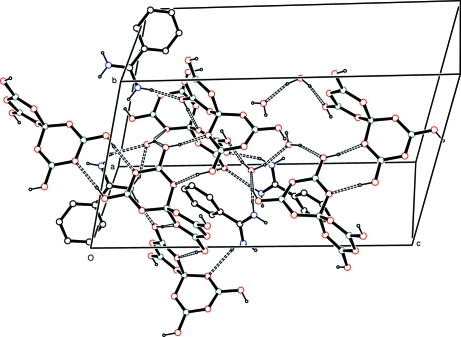
Crystal packing diagram for the title compound viewed approximately down the a axis. All atoms are shown as small spheres of arbitrary radii. For the sake of clarity, H atoms not involved in hydrogen bonding are omitted. Hydrogen bonding is indicated by dashed lines.

## supplementary crystallographic information

### Comment

The asymmetric unit of the title compound (Fig. 1) comprises two planar benzamidinium cations, two tetrahydropentaborate anions and three water molecules of crystallization. The anions consist of a central BO_4_ tetrahedron fused to four trigonal planar BO_3_(OH) units. Both cations and the anions show normal geometric parameters (Table 1). Analysis of the crystal packing (Fig. 2) shows that adjacent [B_5_O_6_(OH)_4_]^-^ units are hydrogen bonded to form eight-membered rings of graph set *R*^2^_2_(8) (Etter *et al.*, 1990; Bernstein *et al.*, 1995; Motherwell *et al.*, 1999). This anion-to-anion hydrogen-bonding framework is supplemented by the formation of five hydrogen bonds from each benzamidinium cation to adjacent [B_5_O_6_(OH)_4_]^-^ anions (Table 2). Interestingly, two of the three water molecules of crystallization form hydrogen bonds which involve all the available hydrogen-bond donor/acceptor sites, at variance with what has been found in a survey of 3315 organic hydrate crystal structures. In this study (Gillon *et al.*, 2003) it has been shown that the most common environment is one in which water forms three hydrogen bonds, two as donor and one as acceptor.

### Experimental

B(OH)_3_ (90 mg, 0.15 mmol, Sigma Aldrich at 99.5% purity) was added to a stirred solution of benzamidine, C_7_H_8_N_2_, (12 mg, 0.1 mmol, Fluka at 95% purity) in water (10 ml) and heated under reflux for 3 h. After cooling the solution to ambient temperature, crystals suitable for single-crystal X-ray diffraction were grown by slow evaporation of the solvent over several days.

### Refinement

All H atoms were found in a difference map. Positional and isotropic parameters of H atoms of the hydroxy and amino groups, as well as positional parameters of H atoms of the water molecules having U_iso_ values equal to 1.5U_eq_(O), were refined. H atoms of the phenyl rings were positioned with idealized geometry and refined isotropically using a riding model (C–H = 0.97 Å), and their U_iso_ values were kept equal to 1.2U_eq_(C). An antibump restrain was introduced in the final calculation to prevent solvent molecules from approaching too close to one another.

### Crystal data

**Table:**

C_7_H_9_N_2_^+^·B_5_H_4_O_10_^–^·1.5H_2_O	*Z* = 4
*M**_r_* = 366.27	*F*_000_ = 756
Triclinic, *P*1	*D*_x_ = 1.535 Mg m^−^^3^
Hall symbol: -P 1	Mo *K*α radiation λ = 0.71073 Å
*a* = 8.22314 (19) Å	Cell parameters from 36413 reflections
*b* = 10.7814 (2) Å	θ = 2.6–32.6º
*c* = 19.1997 (3) Å	µ = 0.14 mm^−^^1^
α = 75.9475 (11)º	*T* = 298 (2) K
β = 85.4458 (16)º	Block, colourless
γ = 73.6979 (13)º	0.15 × 0.12 × 0.10 mm
*V* = 1584.74 (5) Å^3^	

### Data collection

**Table:**

Oxford Diffraction Xcalibur S CCD diffractometer	9063 independent reflections
Radiation source: Enhance (Mo) X-ray source	5688 reflections with *I* > 2σ(*I*)
Monochromator: graphite	*R*_int_ = 0.050
Detector resolution: 16.0696 pixels mm^-1^	θ_max_ = 30.0º
*T* = 298(2) K	θ_min_ = 2.6º
ω and φ scans	*h* = −11→11
Absorption correction: multi-scan(CrysAlis RED; Oxford Diffraction, 2006)	*k* = −15→15
*T*_min_ = 0.975, *T*_max_ = 0.988	*l* = −27→27
142668 measured reflections	

### Refinement

**Table:**

Refinement on *F*^2^	Secondary atom site location: difference Fourier map
Least-squares matrix: full	Hydrogen site location: inferred from neighbouring sites
*R*[*F*^2^ > 2σ(*F*^2^)] = 0.041	H atoms treated by a mixture of independent and constrained refinement
*wR*(*F*^2^) = 0.098	*w* = 1/[σ^2^(*F*_o_^2^) + (0.0514*P*)^2^] where *P* = (*F*_o_^2^ + 2*F*_c_^2^)/3
*S* = 0.99	(Δ/σ)_max_ = 0.001
9063 reflections	Δρ_max_ = 0.23 e Å^−^^3^
542 parameters	Δρ_min_ = −0.22 e Å^−^^3^
1 restraint	Extinction correction: none
Primary atom site location: structure-invariant direct methods	

### Special details

**Table:**

Geometry. All e.s.d.'s (except the e.s.d. in the dihedral angle between two l.s. planes) are estimated using the full covariance matrix. The cell e.s.d.'s are taken into account individually in the estimation of e.s.d.'s in distances, angles and torsion angles; correlations between e.s.d.'s in cell parameters are only used when they are defined by crystal symmetry. An approximate (isotropic) treatment of cell e.s.d.'s is used for estimating e.s.d.'s involving l.s. planes.
Refinement. Refinement of *F*^2^ against ALL reflections. The weighted *R*-factor *wR* and goodness of fit *S* are based on *F*^2^, conventional *R*-factors *R* are based on *F*, with *F* set to zero for negative *F*^2^. The threshold expression of *F*^2^ > σ(*F*^2^) is used only for calculating *R*-factors(gt) *etc*. and is not relevant to the choice of reflections for refinement. *R*-factors based on *F*^2^ are statistically about twice as large as those based on *F*, and *R*- factors based on ALL data will be even larger.

### Fractional atomic coordinates and isotropic or equivalent isotropic displacement parameters (Å^2^)

**Table:**

	*x*	*y*	*z*	*U*_iso_*/*U*_eq_	
B1	0.15368 (18)	0.67509 (13)	0.78017 (7)	0.0240 (3)	
O1	0.18508 (11)	0.67022 (8)	0.85446 (4)	0.0285 (2)	
B2	0.10340 (19)	0.61230 (14)	0.91179 (7)	0.0264 (3)	
O2	0.13937 (14)	0.61947 (10)	0.97783 (5)	0.0411 (3)	
H2	0.088 (2)	0.5797 (18)	1.0104 (10)	0.061 (5)*	
O3	−0.01246 (12)	0.54462 (9)	0.90282 (4)	0.0324 (2)	
O4	−0.14621 (13)	0.46036 (10)	0.82903 (6)	0.0414 (3)	
H4	−0.146 (2)	0.4444 (19)	0.7868 (11)	0.065 (6)*	
B3	−0.03994 (19)	0.53439 (14)	0.83403 (7)	0.0275 (3)	
O5	0.03619 (11)	0.59533 (9)	0.77633 (4)	0.0298 (2)	
O6	0.31411 (11)	0.61782 (8)	0.74602 (4)	0.0276 (2)	
O7	0.49090 (14)	0.60254 (10)	0.64314 (5)	0.0415 (3)	
H7	0.536 (3)	0.521 (2)	0.6692 (12)	0.082 (7)*	
O8	0.25892 (12)	0.78921 (9)	0.63891 (4)	0.0337 (2)	
B4	0.3560 (2)	0.66751 (14)	0.67700 (7)	0.0282 (3)	
O9	0.05933 (13)	0.99063 (9)	0.63637 (5)	0.0370 (2)	
H9	−0.009 (3)	1.037 (2)	0.6630 (11)	0.071 (6)*	
O10	0.08133 (11)	0.81424 (8)	0.74163 (4)	0.0286 (2)	
B5	0.13056 (19)	0.86493 (14)	0.67397 (7)	0.0261 (3)	
B1A	0.20283 (19)	0.70679 (14)	0.31266 (7)	0.0251 (3)	
O1A	0.14676 (11)	0.85043 (8)	0.27918 (4)	0.0294 (2)	
B2A	0.22073 (19)	0.90684 (14)	0.21888 (7)	0.0256 (3)	
O2A	0.16712 (13)	1.03877 (9)	0.18762 (5)	0.0331 (2)	
H2A	0.088 (2)	1.0818 (18)	0.2128 (10)	0.058 (6)*	
O3A	0.35330 (12)	0.83347 (9)	0.18527 (5)	0.0347 (2)	
O4A	0.55788 (14)	0.63416 (10)	0.18374 (5)	0.0400 (3)	
H4A	0.602 (3)	0.551 (2)	0.2064 (11)	0.077 (6)*	
B3A	0.4266 (2)	0.70280 (14)	0.21817 (7)	0.0277 (3)	
O5A	0.36698 (11)	0.64584 (8)	0.28257 (4)	0.0304 (2)	
O6A	0.08059 (12)	0.63966 (9)	0.29702 (5)	0.0338 (2)	
O7A	−0.14440 (18)	0.53852 (17)	0.33468 (7)	0.0770 (5)	
H7A	−0.126 (3)	0.513 (2)	0.2918 (12)	0.094 (7)*	
O8A	−0.04403 (13)	0.63084 (11)	0.41341 (5)	0.0448 (3)	
B4A	−0.0342 (2)	0.60380 (18)	0.34663 (9)	0.0398 (4)	
O9A	0.09860 (16)	0.66666 (12)	0.50576 (5)	0.0491 (3)	
H9A	0.018 (3)	0.641 (2)	0.5317 (11)	0.075 (6)*	
O10A	0.21717 (11)	0.69028 (9)	0.38973 (4)	0.0302 (2)	
B5A	0.0913 (2)	0.66232 (15)	0.43589 (8)	0.0318 (3)	
N1	0.67173 (18)	0.80236 (14)	−0.07944 (6)	0.0402 (3)	
H11	0.631 (2)	0.7525 (17)	−0.0989 (9)	0.051 (5)*	
H12	0.716 (2)	0.8610 (17)	−0.1079 (9)	0.050 (5)*	
N2	0.61716 (17)	0.68505 (12)	0.03102 (7)	0.0385 (3)	
H21	0.581 (2)	0.6316 (16)	0.0105 (9)	0.048 (5)*	
H22	0.599 (2)	0.6779 (16)	0.0763 (10)	0.049 (5)*	
C1	0.73291 (16)	0.87110 (13)	0.02400 (7)	0.0301 (3)	
C2	0.71988 (18)	1.00220 (14)	−0.01273 (8)	0.0370 (3)	
H2B	0.6713	1.0354	−0.0603	0.044*	
C3	0.7765 (2)	1.08450 (15)	0.01889 (9)	0.0471 (4)	
H3	0.7675	1.1756	−0.0066	0.057*	
C4	0.8452 (2)	1.03783 (17)	0.08617 (10)	0.0528 (4)	
H4B	0.8834	1.0965	0.1083	0.063*	
C5	0.8603 (2)	0.90783 (18)	0.12271 (9)	0.0514 (4)	
H5	0.9105	0.8751	0.1700	0.062*	
C6	0.80389 (19)	0.82453 (15)	0.09177 (8)	0.0412 (3)	
H6	0.8139	0.7334	0.1175	0.049*	
C7	0.67193 (16)	0.78329 (13)	−0.00935 (7)	0.0297 (3)	
N1A	0.3078 (2)	0.85153 (14)	0.48086 (8)	0.0550 (4)	
H11A	0.325 (2)	0.8023 (18)	0.4498 (10)	0.058 (5)*	
H12A	0.268 (2)	0.8266 (18)	0.5252 (11)	0.061 (5)*	
N2A	0.2464 (2)	1.06351 (15)	0.49387 (7)	0.0478 (4)	
H21A	0.254 (2)	1.1441 (19)	0.4800 (10)	0.063 (6)*	
H22A	0.193 (2)	1.0430 (17)	0.5356 (10)	0.058 (5)*	
C1A	0.38252 (19)	1.01425 (14)	0.38368 (7)	0.0358 (3)	
C2A	0.5160 (2)	0.92500 (17)	0.35919 (8)	0.0490 (4)	
H23A	0.5592	0.8364	0.3889	0.059*	
C3A	0.5890 (2)	0.96110 (19)	0.29228 (9)	0.0571 (5)	
H3A	0.6822	0.8976	0.2750	0.069*	
C4A	0.5286 (2)	1.08720 (19)	0.25051 (8)	0.0550 (5)	
H41A	0.5811	1.1131	0.2042	0.066*	
C5A	0.3948 (2)	1.17638 (17)	0.27401 (8)	0.0493 (4)	
H5A	0.3524	1.2648	0.2441	0.059*	
C6A	0.3197 (2)	1.14088 (15)	0.34044 (7)	0.0411 (3)	
H6A	0.2240	1.2038	0.3567	0.049*	
C7A	0.30793 (19)	0.97547 (15)	0.45592 (7)	0.0378 (3)	
O1W	0.53676 (17)	0.59539 (14)	−0.10037 (7)	0.0554 (3)	
H11W	0.441 (3)	0.614 (2)	−0.1163 (13)	0.089*	
H12W	0.603 (3)	0.545 (2)	−0.1244 (12)	0.089*	
O2W	−0.25372 (16)	0.66371 (12)	0.54656 (6)	0.0512 (3)	
H21W	−0.296 (3)	0.649 (2)	0.5068 (12)	0.082*	
H22W	−0.335 (3)	0.651 (2)	0.5809 (12)	0.082*	
O3W	−0.41457 (19)	0.58780 (16)	0.44572 (9)	0.0732 (4)	
H31W	−0.492 (4)	0.568 (2)	0.4515 (8)	0.117*	
H32W	−0.329 (3)	0.549 (3)	0.4128 (15)	0.117*	

### Atomic displacement parameters (Å^2^)

**Table:**

	*U*^11^	*U*^22^	*U*^33^	*U*^12^	*U*^13^	*U*^23^
B1	0.0284 (8)	0.0219 (7)	0.0200 (6)	−0.0071 (6)	0.0052 (6)	−0.0030 (5)
O1	0.0352 (5)	0.0315 (5)	0.0218 (4)	−0.0160 (4)	0.0038 (4)	−0.0049 (4)
B2	0.0314 (8)	0.0229 (7)	0.0236 (6)	−0.0070 (6)	0.0053 (6)	−0.0052 (5)
O2	0.0598 (7)	0.0498 (6)	0.0233 (5)	−0.0322 (6)	0.0054 (5)	−0.0078 (4)
O3	0.0402 (5)	0.0402 (5)	0.0224 (4)	−0.0222 (5)	0.0081 (4)	−0.0070 (4)
O4	0.0500 (6)	0.0522 (6)	0.0330 (5)	−0.0315 (5)	0.0091 (5)	−0.0129 (5)
B3	0.0278 (8)	0.0276 (7)	0.0271 (7)	−0.0081 (7)	0.0052 (6)	−0.0071 (6)
O5	0.0369 (5)	0.0338 (5)	0.0222 (4)	−0.0170 (4)	0.0050 (4)	−0.0061 (4)
O6	0.0298 (5)	0.0230 (4)	0.0245 (4)	−0.0036 (4)	0.0067 (4)	−0.0013 (3)
O7	0.0450 (6)	0.0287 (5)	0.0366 (5)	0.0006 (5)	0.0203 (5)	−0.0003 (4)
O8	0.0427 (6)	0.0266 (5)	0.0232 (4)	−0.0019 (4)	0.0090 (4)	−0.0016 (4)
B4	0.0306 (8)	0.0252 (7)	0.0262 (7)	−0.0073 (7)	0.0062 (6)	−0.0038 (6)
O9	0.0433 (6)	0.0262 (5)	0.0293 (5)	0.0011 (5)	0.0089 (4)	0.0009 (4)
O10	0.0313 (5)	0.0230 (4)	0.0251 (4)	−0.0024 (4)	0.0077 (4)	−0.0021 (3)
B5	0.0283 (8)	0.0250 (7)	0.0234 (6)	−0.0065 (7)	0.0033 (6)	−0.0046 (6)
B1A	0.0308 (8)	0.0224 (7)	0.0213 (6)	−0.0086 (6)	0.0070 (6)	−0.0041 (5)
O1A	0.0314 (5)	0.0227 (4)	0.0290 (4)	−0.0046 (4)	0.0097 (4)	−0.0025 (3)
B2A	0.0287 (8)	0.0234 (7)	0.0225 (6)	−0.0061 (7)	0.0020 (6)	−0.0030 (5)
O2A	0.0392 (6)	0.0231 (5)	0.0298 (5)	−0.0035 (5)	0.0113 (4)	−0.0022 (4)
O3A	0.0403 (6)	0.0254 (5)	0.0273 (4)	−0.0006 (4)	0.0132 (4)	0.0008 (4)
O4A	0.0451 (6)	0.0278 (5)	0.0310 (5)	0.0040 (5)	0.0176 (5)	0.0016 (4)
B3A	0.0313 (8)	0.0247 (7)	0.0243 (6)	−0.0062 (7)	0.0064 (6)	−0.0041 (6)
O5A	0.0340 (5)	0.0222 (4)	0.0273 (4)	−0.0031 (4)	0.0108 (4)	−0.0004 (3)
O6A	0.0428 (6)	0.0401 (5)	0.0258 (4)	−0.0227 (5)	0.0096 (4)	−0.0107 (4)
O7A	0.0885 (10)	0.1351 (13)	0.0538 (7)	−0.0873 (10)	0.0356 (7)	−0.0531 (8)
O8A	0.0456 (6)	0.0691 (7)	0.0348 (5)	−0.0343 (6)	0.0169 (5)	−0.0235 (5)
B4A	0.0435 (10)	0.0510 (10)	0.0350 (8)	−0.0250 (8)	0.0115 (7)	−0.0181 (7)
O9A	0.0629 (7)	0.0669 (7)	0.0265 (5)	−0.0346 (6)	0.0105 (5)	−0.0115 (5)
O10A	0.0352 (5)	0.0343 (5)	0.0226 (4)	−0.0143 (4)	0.0043 (4)	−0.0054 (4)
B5A	0.0396 (9)	0.0303 (8)	0.0263 (7)	−0.0122 (7)	0.0066 (6)	−0.0066 (6)
N1	0.0556 (8)	0.0442 (7)	0.0269 (6)	−0.0247 (7)	0.0057 (6)	−0.0083 (5)
N2	0.0525 (8)	0.0360 (7)	0.0302 (6)	−0.0198 (6)	0.0052 (6)	−0.0062 (5)
C1	0.0297 (7)	0.0317 (7)	0.0289 (6)	−0.0092 (6)	0.0054 (5)	−0.0079 (5)
C2	0.0374 (8)	0.0330 (7)	0.0385 (7)	−0.0088 (6)	0.0037 (6)	−0.0065 (6)
C3	0.0453 (9)	0.0326 (8)	0.0653 (11)	−0.0120 (7)	0.0026 (8)	−0.0141 (7)
C4	0.0461 (10)	0.0553 (10)	0.0698 (11)	−0.0189 (8)	0.0000 (8)	−0.0323 (9)
C5	0.0511 (10)	0.0648 (11)	0.0447 (9)	−0.0196 (9)	−0.0080 (7)	−0.0175 (8)
C6	0.0459 (9)	0.0409 (8)	0.0370 (7)	−0.0136 (7)	−0.0034 (6)	−0.0062 (6)
C7	0.0295 (7)	0.0286 (7)	0.0286 (6)	−0.0062 (6)	0.0039 (5)	−0.0055 (5)
N1A	0.0941 (12)	0.0436 (8)	0.0342 (7)	−0.0350 (8)	0.0173 (8)	−0.0086 (6)
N2A	0.0679 (10)	0.0420 (8)	0.0358 (7)	−0.0226 (7)	0.0164 (7)	−0.0096 (6)
C1A	0.0457 (9)	0.0414 (8)	0.0259 (6)	−0.0220 (8)	0.0011 (6)	−0.0067 (6)
C2A	0.0588 (11)	0.0464 (9)	0.0380 (8)	−0.0137 (9)	0.0055 (8)	−0.0050 (7)
C3A	0.0572 (11)	0.0706 (12)	0.0416 (9)	−0.0162 (10)	0.0106 (8)	−0.0142 (8)
C4A	0.0590 (11)	0.0799 (13)	0.0296 (7)	−0.0331 (11)	0.0037 (7)	−0.0037 (8)
C5A	0.0600 (11)	0.0554 (10)	0.0319 (7)	−0.0250 (9)	−0.0059 (7)	0.0039 (7)
C6A	0.0472 (9)	0.0451 (8)	0.0332 (7)	−0.0181 (7)	−0.0022 (6)	−0.0058 (6)
C7A	0.0462 (9)	0.0422 (8)	0.0293 (7)	−0.0219 (7)	0.0014 (6)	−0.0055 (6)
O1W	0.0458 (7)	0.0704 (9)	0.0604 (8)	−0.0210 (7)	−0.0036 (6)	−0.0271 (6)
O2W	0.0620 (8)	0.0562 (7)	0.0451 (6)	−0.0315 (6)	0.0243 (6)	−0.0193 (5)
O3W	0.0637 (10)	0.0796 (10)	0.0819 (10)	−0.0187 (8)	0.0071 (8)	−0.0325 (8)

### Geometric parameters (Å, °)

**Table:**

B1—O1	1.4555 (15)	N1—H11	0.870 (18)
B1—O6	1.4674 (17)	N1—H12	0.872 (18)
B1—O10	1.4771 (15)	N2—C7	1.3188 (16)
B1—O5	1.4788 (16)	N2—H21	0.893 (17)
O1—B2	1.3553 (15)	N2—H22	0.858 (17)
B2—O2	1.3494 (16)	C1—C6	1.3874 (19)
B2—O3	1.3955 (16)	C1—C2	1.3962 (18)
O2—H2	0.83 (2)	C1—C7	1.4729 (18)
O3—B3	1.3929 (16)	C2—C3	1.382 (2)
O4—B3	1.3608 (17)	C2—H2B	0.9700
O4—H4	0.87 (2)	C3—C4	1.372 (2)
B3—O5	1.3484 (16)	C3—H3	0.9700
O6—B4	1.3593 (16)	C4—C5	1.382 (2)
O7—B4	1.3472 (19)	C4—H4B	0.9700
O7—H7	0.89 (2)	C5—C6	1.383 (2)
O8—B5	1.3798 (18)	C5—H5	0.9700
O8—B4	1.3900 (17)	C6—H6	0.9700
O9—B5	1.3592 (17)	N1A—C7A	1.3057 (19)
O9—H9	0.87 (2)	N1A—H11A	0.869 (19)
O10—B5	1.3588 (15)	N1A—H12A	0.892 (19)
B1A—O10A	1.4572 (15)	N2A—C7A	1.302 (2)
B1A—O5A	1.4664 (18)	N2A—H21A	0.86 (2)
B1A—O6A	1.4785 (16)	N2A—H22A	0.891 (19)
B1A—O1A	1.4788 (16)	C1A—C2A	1.377 (2)
O1A—B2A	1.3544 (16)	C1A—C6A	1.394 (2)
B2A—O2A	1.3613 (16)	C1A—C7A	1.4839 (18)
B2A—O3A	1.3721 (18)	C2A—C3A	1.389 (2)
O2A—H2A	0.87 (2)	C2A—H23A	0.9700
O3A—B3A	1.3778 (17)	C3A—C4A	1.376 (3)
O4A—B3A	1.3514 (19)	C3A—H3A	0.9700
O4A—H4A	0.89 (2)	C4A—C5A	1.370 (3)
B3A—O5A	1.3601 (16)	C4A—H41A	0.9700
O6A—B4A	1.3533 (18)	C5A—C6A	1.388 (2)
O7A—B4A	1.3559 (19)	C5A—H5A	0.9700
O7A—H7A	0.92 (2)	C6A—H6A	0.9700
O8A—B4A	1.3737 (18)	O1W—H11W	0.82 (2)
O8A—B5A	1.3781 (18)	O1W—H12W	0.85 (2)
O9A—B5A	1.3604 (18)	O2W—H21W	0.93 (2)
O9A—H9A	0.87 (2)	O2W—H22W	0.91 (2)
O10A—B5A	1.3592 (17)	O3W—H31W	0.72 (3)
N1—C7	1.3110 (17)	O3W—H32W	0.98 (3)
			
O1—B1—O6	109.01 (10)	C7—N1—H12	122.4 (11)
O1—B1—O10	109.67 (10)	H11—N1—H12	117.7 (16)
O6—B1—O10	110.02 (9)	C7—N2—H21	119.9 (11)
O1—B1—O5	110.83 (9)	C7—N2—H22	121.9 (11)
O6—B1—O5	107.59 (10)	H21—N2—H22	117.4 (15)
O10—B1—O5	109.70 (10)	C6—C1—C2	119.52 (13)
B2—O1—B1	124.25 (10)	C6—C1—C7	120.52 (12)
O2—B2—O1	118.37 (12)	C2—C1—C7	119.97 (12)
O2—B2—O3	120.85 (11)	C3—C2—C1	119.80 (14)
O1—B2—O3	120.77 (11)	C3—C2—H2B	120.1
B2—O2—H2	113.3 (13)	C1—C2—H2B	120.1
B3—O3—B2	118.95 (10)	C4—C3—C2	120.24 (14)
B3—O4—H4	113.7 (13)	C4—C3—H3	119.9
O5—B3—O4	122.84 (12)	C2—C3—H3	119.9
O5—B3—O3	120.79 (11)	C3—C4—C5	120.45 (14)
O4—B3—O3	116.37 (11)	C3—C4—H4B	119.8
B3—O5—B1	124.05 (10)	C5—C4—H4B	119.8
B4—O6—B1	123.03 (10)	C4—C5—C6	119.90 (15)
B4—O7—H7	111.2 (13)	C4—C5—H5	120.0
B5—O8—B4	119.26 (10)	C6—C5—H5	120.0
O7—B4—O6	122.12 (12)	C5—C6—C1	120.09 (14)
O7—B4—O8	117.66 (11)	C5—C6—H6	120.0
O6—B4—O8	120.22 (13)	C1—C6—H6	120.0
B5—O9—H9	111.6 (13)	N1—C7—N2	119.55 (13)
B5—O10—B1	122.74 (11)	N1—C7—C1	120.16 (12)
O10—B5—O9	123.50 (13)	N2—C7—C1	120.28 (12)
O10—B5—O8	120.89 (11)	C7A—N1A—H11A	116.7 (12)
O9—B5—O8	115.61 (11)	C7A—N1A—H12A	119.3 (12)
O10A—B1A—O5A	109.86 (11)	H11A—N1A—H12A	122.5 (17)
O10A—B1A—O6A	111.11 (9)	C7A—N2A—H21A	122.3 (13)
O5A—B1A—O6A	106.98 (10)	C7A—N2A—H22A	121.0 (11)
O10A—B1A—O1A	108.47 (10)	H21A—N2A—H22A	116.7 (17)
O5A—B1A—O1A	110.50 (9)	C2A—C1A—C6A	119.51 (13)
O6A—B1A—O1A	109.93 (11)	C2A—C1A—C7A	119.90 (13)
B2A—O1A—B1A	122.37 (10)	C6A—C1A—C7A	120.59 (14)
O1A—B2A—O2A	122.20 (13)	C1A—C2A—C3A	120.39 (16)
O1A—B2A—O3A	121.68 (11)	C1A—C2A—H23A	119.8
O2A—B2A—O3A	116.12 (11)	C3A—C2A—H23A	119.8
B2A—O2A—H2A	112.4 (12)	C4A—C3A—C2A	119.79 (18)
B2A—O3A—B3A	119.19 (10)	C4A—C3A—H3A	120.1
B3A—O4A—H4A	115.1 (14)	C2A—C3A—H3A	120.1
O4A—B3A—O5A	121.78 (12)	C5A—C4A—C3A	120.32 (15)
O4A—B3A—O3A	117.46 (11)	C5A—C4A—H41A	119.8
O5A—B3A—O3A	120.76 (13)	C3A—C4A—H41A	119.8
B3A—O5A—B1A	122.46 (10)	C4A—C5A—C6A	120.38 (15)
B4A—O6A—B1A	121.68 (10)	C4A—C5A—H5A	119.8
B4A—O7A—H7A	113.7 (14)	C6A—C5A—H5A	119.8
B4A—O8A—B5A	118.13 (11)	C5A—C6A—C1A	119.59 (16)
O6A—B4A—O7A	123.06 (13)	C5A—C6A—H6A	120.2
O6A—B4A—O8A	121.13 (13)	C1A—C6A—H6A	120.2
O7A—B4A—O8A	115.80 (13)	N2A—C7A—N1A	120.97 (14)
B5A—O9A—H9A	112.3 (13)	N2A—C7A—C1A	120.29 (13)
B5A—O10A—B1A	121.15 (11)	N1A—C7A—C1A	118.74 (14)
O10A—B5A—O9A	119.12 (13)	H11W—O1W—H12W	107 (2)
O10A—B5A—O8A	121.56 (12)	H21W—O2W—H22W	102.8 (18)
O9A—B5A—O8A	119.33 (12)	H31W—O3W—H32W	118 (3)
C7—N1—H11	119.8 (11)		
			
O6—B1—O1—B2	−124.83 (12)	O6A—B1A—O5A—B3A	99.35 (13)
O10—B1—O1—B2	114.67 (13)	O1A—B1A—O5A—B3A	−20.30 (16)
O5—B1—O1—B2	−6.59 (17)	O10A—B1A—O6A—B4A	17.60 (18)
B1—O1—B2—O2	−177.28 (12)	O5A—B1A—O6A—B4A	137.51 (13)
B1—O1—B2—O3	4.01 (19)	O1A—B1A—O6A—B4A	−102.48 (14)
O2—B2—O3—B3	−177.04 (12)	B1A—O6A—B4A—O7A	−177.91 (16)
O1—B2—O3—B3	1.64 (19)	B1A—O6A—B4A—O8A	1.3 (2)
B2—O3—B3—O5	−3.89 (19)	B5A—O8A—B4A—O6A	−17.0 (2)
B2—O3—B3—O4	176.05 (12)	B5A—O8A—B4A—O7A	162.24 (15)
O4—B3—O5—B1	−179.28 (12)	O5A—B1A—O10A—B5A	−140.66 (12)
O3—B3—O5—B1	0.7 (2)	O6A—B1A—O10A—B5A	−22.48 (17)
O1—B1—O5—B3	4.27 (17)	O1A—B1A—O10A—B5A	98.46 (13)
O6—B1—O5—B3	123.36 (12)	B1A—O10A—B5A—O9A	−170.58 (12)
O10—B1—O5—B3	−116.98 (13)	B1A—O10A—B5A—O8A	8.9 (2)
O1—B1—O6—B4	−141.66 (11)	B4A—O8A—B5A—O10A	12.0 (2)
O10—B1—O6—B4	−21.37 (16)	B4A—O8A—B5A—O9A	−168.55 (14)
O5—B1—O6—B4	98.08 (13)	C6—C1—C2—C3	−0.5 (2)
B1—O6—B4—O7	−169.70 (12)	C7—C1—C2—C3	179.67 (13)
B1—O6—B4—O8	10.48 (19)	C1—C2—C3—C4	0.0 (2)
B5—O8—B4—O7	−173.33 (12)	C2—C3—C4—C5	0.7 (3)
B5—O8—B4—O6	6.49 (19)	C3—C4—C5—C6	−0.9 (3)
O1—B1—O10—B5	137.70 (12)	C4—C5—C6—C1	0.3 (2)
O6—B1—O10—B5	17.80 (16)	C2—C1—C6—C5	0.4 (2)
O5—B1—O10—B5	−100.36 (13)	C7—C1—C6—C5	−179.81 (14)
B1—O10—B5—O9	176.52 (12)	C6—C1—C7—N1	−151.51 (14)
B1—O10—B5—O8	−3.40 (19)	C2—C1—C7—N1	28.3 (2)
B4—O8—B5—O10	−9.94 (19)	C6—C1—C7—N2	28.5 (2)
B4—O8—B5—O9	170.13 (12)	C2—C1—C7—N2	−151.66 (13)
O10A—B1A—O1A—B2A	133.89 (12)	C6A—C1A—C2A—C3A	0.8 (2)
O5A—B1A—O1A—B2A	13.41 (16)	C7A—C1A—C2A—C3A	−178.70 (15)
O6A—B1A—O1A—B2A	−104.44 (13)	C1A—C2A—C3A—C4A	0.6 (3)
B1A—O1A—B2A—O2A	179.00 (11)	C2A—C3A—C4A—C5A	−1.3 (3)
B1A—O1A—B2A—O3A	−0.22 (19)	C3A—C4A—C5A—C6A	0.5 (3)
O1A—B2A—O3A—B3A	−8.04 (19)	C4A—C5A—C6A—C1A	0.8 (2)
O2A—B2A—O3A—B3A	172.69 (11)	C2A—C1A—C6A—C5A	−1.5 (2)
B2A—O3A—B3A—O4A	−179.36 (12)	C7A—C1A—C6A—C5A	177.97 (13)
B2A—O3A—B3A—O5A	1.16 (19)	C2A—C1A—C7A—N2A	147.16 (16)
O4A—B3A—O5A—B1A	−165.33 (12)	C6A—C1A—C7A—N2A	−32.3 (2)
O3A—B3A—O5A—B1A	14.13 (19)	C2A—C1A—C7A—N1A	−31.8 (2)
O10A—B1A—O5A—B3A	−139.94 (12)	C6A—C1A—C7A—N1A	148.75 (15)

### Hydrogen-bond geometry (Å, °)

**Table:**

*D*—H···*A*	*D*—H	H···*A*	*D*···*A*	*D*—H···*A*
O2—H2···O3^i^	0.83 (2)	2.04 (2)	2.8562 (13)	167.4 (18)
O4—H4···O6A^ii^	0.87 (2)	2.00 (2)	2.8361 (13)	161.3 (18)
O7—H7···O5A^iii^	0.89 (2)	1.80 (2)	2.6877 (13)	177 (2)
O9—H9···O1A^iv^	0.87 (2)	1.91 (2)	2.7784 (14)	174.6 (19)
O2A—H2A···O10^iv^	0.87 (2)	1.84 (2)	2.7050 (14)	174.5 (17)
O4A—H4A···O6^iii^	0.89 (2)	1.79 (2)	2.6735 (13)	178 (2)
O7A—H7A···O5^ii^	0.92 (2)	1.93 (2)	2.8085 (15)	160 (2)
O9A—H9A···O2W	0.87 (2)	2.18 (2)	2.9474 (16)	147.4 (18)
N1—H11···O1W	0.870 (18)	2.059 (18)	2.8756 (18)	156.0 (16)
N1—H12···O2A^v^	0.872 (18)	1.996 (18)	2.8484 (15)	165.7 (15)
N2—H21···O1W	0.893 (17)	2.330 (17)	3.0892 (18)	142.8 (14)
N2—H22···O4A	0.858 (17)	2.025 (18)	2.8772 (16)	172.0 (15)
N1A—H11A···O10A	0.869 (19)	2.238 (19)	3.0084 (18)	147.7 (16)
N1A—H12A···O8	0.892 (19)	2.12 (2)	2.9646 (17)	157.4 (16)
N2A—H21A···O2W^iv^	0.86 (2)	2.01 (2)	2.8703 (18)	175.0 (18)
N2A—H22A···O9	0.891 (19)	2.181 (19)	3.0717 (16)	178.4 (16)
O1W—H11W···O1^vi^	0.82 (2)	2.10 (2)	2.9180 (15)	173 (2)
O1W—H12W···O4^vii^	0.85 (2)	2.22 (2)	3.0033 (18)	153 (2)
O2W—H21W···O3W	0.93 (2)	1.92 (2)	2.8199 (19)	163 (2)
O2W—H22W···O7^viii^	0.91 (2)	1.90 (2)	2.8016 (14)	169.3 (19)
O3W—H31W···O3W^ix^	0.72 (3)	2.488 (10)	3.003 (3)	130.5 (17)
O3W—H32W···O7A	0.98 (3)	2.05 (3)	2.983 (2)	159 (2)

Symmetry codes: (i) −*x*, −*y*+1, −*z*+2; (ii) −*x*, −*y*+1, −*z*+1; (iii) −*x*+1, −*y*+1, −*z*+1; (iv) −*x*, −*y*+2, −*z*+1; (v) −*x*+1, −*y*+2, −*z*; (vi) *x*, *y*, *z*−1; (vii) *x*+1, *y*, *z*−1; (viii) *x*−1, *y*, *z*; (ix) −*x*−1, −*y*+1, −*z*+1.
